# Supplementary material for: Splice-Junction-Based Mapping of Alternative Isoforms in the Human Proteome
Source: Cell Rep. Author manuscript; Available in PMC 2020 Jan 15. (PMC6961840; doi:10.1016/j.celrep.2019.11.026)
Supplement: 3 [file NIHMS1546469-supplement-3.zip › DF2/PXD0010154/Liver-172-Q99959-QITGLLWNLSSNDKLK.pdf]

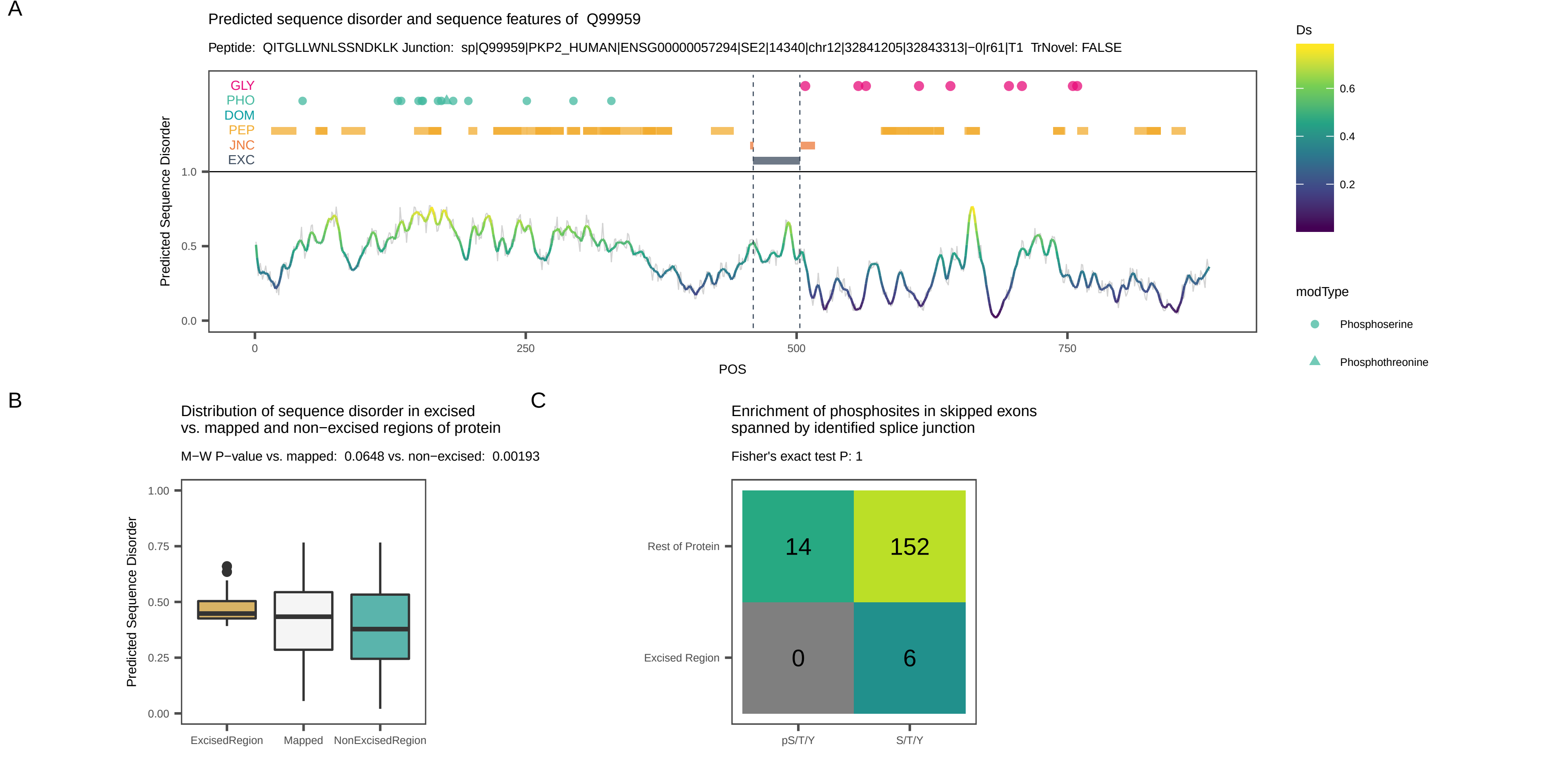
A
Predicted sequence disorder and sequence features of Q99959
Peptide: QITGLLWNLSSNDKLK Junction: sp|Q99959|PKP2_HUMAN|ENSG00000057294|SE2|14340|chr12|32841205|32843313|−0|r61|T1 TrNovel: FALSE
GLY
PHO
DOM
PEP
JNC
EXC
1.0
0.5
0.0
Predicted Sequence Disorder
0
250
500
750
POS
Ds
0.6
0.4
0.2
modType
Phosphoserine
Phosphothreonine
B
Distribution of sequence disorder in excised
vs. mapped and non−excised regions of protein
M−W P−value vs. mapped: 0.0648 vs. non−excised: 0.00193
1.00
0.75
0.50
0.25
0.00
Predicted Sequence Disorder
ExcisedRegion
Mapped
NonExcisedRegion
C
Enrichment of phosphosites in skipped exons
spanned by identified splice junction
Fisher's exact test P: 1
Rest of Protein
Excised Region
14
152
0
6
pS/T/Y
S/T/Y
